# Tendência temporal da prevalência de desnutrição em crianças menores de 5 anos assistidas pelo Programa Bolsa Família (2008-2019) 

**DOI:** 10.1590/0102-311XPT180022

**Published:** 2024-02-09

**Authors:** Allan Victor da Silveira Gouveia, Renata Elyonara de Sousa Carvalho, Maria Eduarda Guimarães Correia, Jonas Augusto Cardoso da Silveira

**Affiliations:** 1 Programa de Pós-graduação em Nutrição, Universidade Federal de Alagoas, Maceió, Brasil.; 2 Departamento de Nutrição, Universidade Federal do Paraná, Curitiba, Brasil.

**Keywords:** Criança, Desnutrição, Vigilância Alimentar e Nutricional, Desigualdade em Saúde, Child, Malnutrition, Food and Nutritional Surveillance, Health Status Disparities, Niño, Desnutrición, Vigilancia Alimentaria y Nutricional, Desigualdad en Salud

## Abstract

O objetivo deste estudo foi analisar a tendência temporal da desnutrição em crianças menores de 5 anos de idade assistidas pelo Programa Bolsa Família entre 2008 e 2019, explorando desigualdades regionais e buscando determinar o impacto das crises econômica e política agravadas em 2014 e da adesão governamental às políticas de austeridade fiscal na tendência. As análises foram realizadas utilizando dados agregados de lactentes (0-23 meses) e pré-escolares (24-59 meses), extraídos do Sistema de Vigilância Alimentar e Nutricional (SISVAN) assistidas pelo Programa Bolsa Família (n = 34.272.024). As tendências foram analisadas por meio de modelos lineares generalizados, com efeitos mistos específicos para as faixas etárias (distribuição binomial negativa e função de ligação log). As desigualdades regionais foram analisadas a partir do agrupamento das Unidades Federativas segundo o Índice de Vulnerabilidade Social (IVS) e a influência das crises e das políticas de austeridade na prevalência de desnutrição por meio da interação entre “ano” e “crise” (2008-2013 *vs.* 2014-2019). Houve redução na prevalência de desnutrição infantil até meados de 2013, quando as tendências passaram a ser estacionárias para pré-escolares e ascendentes para lactentes. Observou-se, também, maior risco de desnutrição nos estados com média e alta vulnerabilidade social, quando comparadas àqueles com baixa vulnerabilidade social. Os pontos de inflexão nas tendências corroboram a hipótese de que as crises política e econômica, e as respostas governamentais a essas crises, provocaram impacto negativo sobre o estado nutricional de crianças em situação de pobreza e extrema pobreza no Brasil.

## Abstract (en)

This study aimed to analyze the temporal trend of malnutrition in children aged under five years assisted by the Brazilian Income Transfer Program from 2008 to 2019, by exploring regional inequalities and seeking to determine the impact of the economic and political crises aggravated in 2014, and the government’s adherence to fiscal austerity policies on the trend. The analyses were performed using aggregated data from infants (0-23 months) and preschoolers (24-59 months), extracted from the Brazilian Food and Nutritional Surveillance System (SISVAN) assisted by the Brazilian Income Transfer Program (n = 34,272,024). Trends were analyzed using generalized linear models with age-specific mixed effects (negative binomial distribution and log linkage function). The regional inequalities were analyzed based on the grouping of Federative Units according to the Social Vulnerability Index (SVI) and the influence of crises and austerity policies on the prevalence of malnutrition by the interaction between “year” and “crisis” (2008-2013 vs. 2014-2019). There was a reduction in the prevalence of child malnutrition until mid-2013, when the trends became stationary for preschoolers and upward for infants. There was also a higher risk of malnutrition in Federative Units with medium- and high-social vulnerability, when compared to those with low-social vulnerability. The inflection points in the trends corroborate the hypothesis that the political and economic crises, and the governmental responses to these crises, negatively impacted the nutritional status of children in poverty and extreme poverty in Brazil.

## Abstract (es)

El objetivo de este estudio fue analizar la tendencia temporal de la desnutrición en niños menores de cinco años atendidos por el Programa Bolsa Familia entre los años 2008 y 2019, explorando desigualdades regionales y buscando determinar el impacto de las crisis económica y política que se intensificaron en 2014, así como la adhesión del gobierno a políticas de austeridad fiscal en esta tendencia. Los análisis se realizaron utilizando datos agregados de lactantes (0-23 meses) y preescolares (24-59 meses), extraídos del Sistema de Vigilancia Alimentaria y Nutricional (SISVAN) atendidos por el Programa Bolsa Familia (n = 34.272.024). Se analizaron las tendencias a través de modelos lineales generalizados con efectos mixtos específicos para los grupos de edad (distribución binomial negativa y función de enlace de logaritmo). Se analizaron las desigualdades regionales a partir del agrupamiento de las unidades federativas conforme el Índice de Vulnerabilidad Social (IVS) y la influencia de las crisis y de las políticas de austeridad en la prevalencia de desnutrición a través de la interacción entre “año” y “crisis” (2008-2013 vs. 2014-2019). Hubo una disminución en la prevalencia de desnutrición infantil hasta mediados de 2013, cuando las tendencias se volvieron estacionarias para preescolares y ascendentes para lactantes. También se observó un riesgo más alto de desnutrición en estados con vulnerabilidad social media y alta, en comparación con aquellos con vulnerabilidad social baja. Los puntos de inflexión en las tendencias corroboran la hipótesis de que las crisis política y económica, y las respuestas del gobierno para estas crisis, tuvieron un impacto negativo en el estado nutricional de niños en situación de pobreza y extrema pobreza en Brasil.

## Introdução

Condições ambientais inapropriadas durante a infância, principalmente em decorrência da pobreza, são determinantes no comprometimento do potencial de crescimento e desenvolvimento dos indivíduos ^1^^,^^2^. As consequências, em curto, médio e longo prazos, desse comprometimento se desvelam desde atrasos no desenvolvimento neuropsicomotor, menor desempenho acadêmico, dificuldades na autorregulação comportamental e emocional, até aumento da morbimortalidade por causas evitáveis e redução do capital humano, da produtividade e dos anos de vida ajustados pela qualidade ^1^^,^^2^^,^^3^^,^^4^^,^^5^.

Por essas razões, os Estados-membros da Organização das Nações Unidas (ONU) formalizaram compromissos para a erradicação da pobreza e da fome, como os Objetivos de Desenvolvimento do Milênio (ODM) ^6^ e, posteriormente, os Objetivos de Desenvolvimento Sustentável (ODS) ^7^. Em resposta a esses compromissos, a prevalência mundial de baixa estatura para idade em crianças < 5 anos caiu de 39,7%, em 1990, para 22,9%, em 2016 ^8^. Entretanto, apesar do declínio sistemático na desnutrição infantil em nível global, a prevalência ainda permanece aproximadamente dez vezes acima do esperado, concentrando-se, majoritariamente, em países mais desiguais e com menor Índice de Desenvolvimento Humano (IDH) ^9^.

No Brasil, a priorização de uma agenda interministerial de segurança alimentar e nutricional, os avanços das políticas públicas de suporte social e o crescimento econômico até o ano de 2013 (associado ao aumento real dos ganhos familiares) produziram redução da prevalência de desnutrição infantil. Nesse período, apesar das desigualdades sociais e econômicas ainda existirem, observou-se significativa redução das iniquidades em saúde, devido à expressiva redução da desnutrição entre crianças vivendo em situação de pobreza ^1^^,^^2^^,^^8^^,^^10^. Entre 1975 e 2006, inquéritos populacionais demonstraram que a prevalência de baixa estatura reduziu de 37,1% para 7,1% e as razões de prevalências entre as crianças mais pobres e mais ricas passou de 4,9 para 2,6 vezes ^1^.

Nesse contexto, em 2004, surge o Programa Bolsa Família como uma das principais estratégias de superação da pobreza e da extrema pobreza, condições definidas pela *Lei nº 10.836/2004*^11^ e atualizadas pelo *Decreto nº 8.794/2016*^12^ como R$ 170,00 e R$ 85,00, respectivamente, no intuito de proporcionar às família meios de acessar direitos básicos pela transferência direta e condicional de renda ^13^.

Extinto pelo *Decreto nº 10.852/2021*^14^, o Programa Bolsa Família foi o maior programa de transferência condicional de renda do mundo, alcançando cerca de 19,5 milhões de famílias, sendo o ponto chave para a retirada do Brasil do mapa da fome no ano de 2014 ^13^, quando se atingiu a menor prevalência de insegurança alimentar em toda a série histórica (22,9%) ^15^.

No entanto, a eclosão das crises política e econômica no país, em 2014, e a adoção de profundas medidas de austeridade fiscal em resposta a essas crises produziram importantes barreiras para a garantia do direito humano à alimentação e nutrição adequadas (DHANA) da população brasileira. Associado à progressiva desarticulação do Sistema Nacional de Segurança Alimentar e Nutricional (SISAN), já em 2018, a prevalência de famílias em insegurança alimentar no Brasil aumentou para 36,6%, retornando ao mesmo patamar de 2014 (35,3%). Diante desse cenário de desmonte do estado de bem-estar social e da emergência da crise sanitária da COVID-19, em 2020, observou-se que 55,2% das famílias brasileiras foram submetidas à situação de insegurança alimentar ^16^^,^^17^.

Dessa forma, faz-se fundamental monitorar o perfil nutricional de crianças em situação de vulnerabilidade socioeconômica, a fim de compreender o cenário epidemiológico atual e subsidiar a gestão de políticas públicas e o trabalho de organizações da sociedade civil de defesa da infância e do DHANA.

O Sistema de Vigilância Alimentar e Nutricional (SISVAN) é responsável pelo monitoramento do estado nutricional e do consumo alimentar de pessoas acompanhadas na atenção primária à saúde (APS), sendo o pilar do ciclo de vigilância alimentar e nutricional no Brasil, sobretudo por proporcionar a estratificação por áreas, tendo em vista a dimensão continental e de padrão de desenvolvimento econômico e social desigual existente entre as Unidades Federativas (UF) do país ^18^. É um sistema que possibilita a integração do Sistema Único de Saúde (SUS) com o SISAN e o Sistema Único de Assistência Social (SUAS) ^16^.

Diante do cenário de desmonte das políticas de segurança alimentar e nutricional e de violações do DHANA, o objetivo deste trabalho foi analisar a tendência temporal da prevalência de desnutrição em crianças < 5 anos de idade assistidas pelo Programa Bolsa Família, explorando desigualdades regionais a partir do agrupamento das UF segundo o Índice de Vulnerabilidade Social (IVS). Além disso, buscou-se determinar o impacto das crises econômica e política e da adesão governamental às políticas de austeridade fiscal na tendência.

## Métodos

### Desenho do estudo

Este é um estudo ecológico construído a partir de relatórios públicos do SISVAN de 2008 a 2019, selecionando todas as crianças < 5 anos cadastradas no módulo do Sistema de Gestão do Bolsa Família. Os dados foram extraídos de forma agregada e em formato de contagem (número de crianças com o evento e a população total), estratificados por faixa etária (menores que 24 meses e 24 a 59 meses) e por UF (26 estados brasileiros e o Distrito Federal), por meio do Sistema e-Gestor da atenção básica (http://sisaps.saude.gov.br/sisvan/ - acessado em 22/Fev/2022).

Uma vez que o acompanhamento do estado nutricional pelas unidades de saúde era uma das condicionalidades para a transferência de renda no Programa Bolsa Família ^1^, os dados extraídos do SISVAN apresentam característica censitária dessa população. Em média, foram acompanhados anualmente 612.971 lactentes e 2.243.031 pré-escolares, totalizando 34.272.024 observações no período de 2008 a 2019. Devido à baixa cobertura e/ou à ausência de eventos de desnutrição em lactentes do Distrito Federal dos anos de 2008 a 2012, essas observações foram desconsideradas nas análises.

### Variáveis estudadas

As prevalências de baixa estatura para idade, baixo peso para idade ou magreza foram definidas, respectivamente, quando os escores-Z dos índices de estatura para idade (E-I), peso para idade (P-I) ou índice de massa corporal para idade (IMC-I) eram ≤ -2 desvios padrão (DP), segundo as curvas de crescimento da Organização Mundial da Saúde (OMS) ^19^.

Os pontos de corte adotados para esses indicadores antropométricos representam diferentes expressões da desnutrição, caracterizada como a ingestão insuficiente de energia e nutrientes (acesso insuficiente aos alimentos/insegurança alimentar), do aumento do gasto energético ou das demandas de nutrientes (geralmente, associadas a processos infecciosos), ou comprometimentos na capacidade de absorção dos nutrientes pelo organismo (digestibilidade da dieta ou doenças relacionadas com o trato gastrointestinal) - processos que não são mutuamente exclusivos. Brevemente, enquanto a baixa estatura (E-I) reflete em inadequações nutricionais crônicas sobre o crescimento linear, o baixo peso (P-I) e a magreza (IMC-I) são sensíveis ao efeito de inadequações em curto prazo, além do efeito cumulativo dessas privações ^20^. Do ponto de vista da avaliação populacional, prevalências de magreza e baixa estatura, respectivamente, acima de 5% e 10% representam problemas importantes para a saúde pública ^21^. Dessa forma, como o foco do trabalho é analisar os déficits nutricionais como um processo social, resultante de uma conjuntura política e econômica, para efeito de discussão, optamos por utilizar a expressão “desnutrição infantil” para abordar esse fenômeno de maneira ampliada, sem endereçar de maneira específica cada um dos três índices, tendo em vista que é constante sua associação com a pobreza e as vulnerabilidades vivenciadas pela população-alvo do estudo.

Apesar de ser uma abordagem tradicional, a agregação das crianças < 5 anos (lactentes e pré-escolares) em um único grupo pode mascarar efeitos ambientais ou de políticas públicas sensíveis às condições de alimentação e nutrição infantil, pois são períodos cujas recomendações alimentares, o grau de desenvolvimento e de autonomia da criança para as escolhas alimentares e as experiências além do ambiente alimentar domiciliar são fundamentalmente diferentes ^22^. Sendo assim, optamos por estratificar as análises pelas faixas etárias de lactentes (< 24 meses) e pré-escolares (24-59 meses).

**Figura 1:**
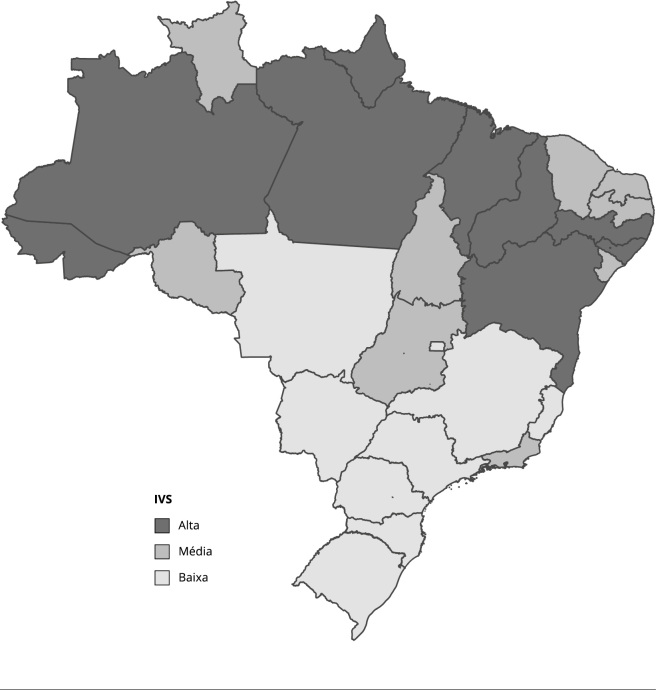
Classificação das Unidades Federativas brasileiras de acordo com o Índice de Vulnerabilidade Social (IVS) para o ano de 2010.

A fim de examinar a influência das desigualdades na prevalência dos diferentes indicadores de desnutrição infantil, as 27 UF do Brasil foram agrupadas em três categorias, segundo o IVS ^23^, sendo elas: (i) muito baixa e baixa vulnerabilidade social (IVS < 0,300); (ii) média vulnerabilidade social (IVS ≥ 0,300 e < 0,400); e (iii) muito alta e alta vulnerabilidade social (IVS ≥ 0,400) (Figura 1). O IVS é um índice multidimensional da situação socioeconômica de uma população, construído a partir de 16 indicadores extraídos do *Censo Demográfico* de 2010 e organizados em três dimensões: infraestrutura urbana; capital humano; e renda e trabalho. Esse índice foi escolhido por indicar a suficiência ou não das ações governamentais no território brasileiro e incorporar diferentes determinantes subjacentes da desnutrição infantil ^24^^,^^25^.

Por último, além do ano do inquérito, criamos a variável dicotômica “crise” (pré-2014 = 0; pós-2014 = 1), a fim de testar o efeito das crises política e econômica e da consequente adesão por políticas de austeridade, que ocorreram a partir de 2014, na prevalência de desnutrição em crianças assistidas pelo Programa Bolsa Família.

### Análise estatística

As séries temporais para cada um dos indicadores de desnutrição foram geradas por meio de modelos lineares generalizados com efeitos mistos específicos para as faixas etárias (distribuição binomial negativa e função de ligação *log*). Optou-se pela distribuição binomial negativa pelo fato de os dados terem sido extraídos como contagem, e as análises iniciais indicarem excesso de variabilidade na prevalência de desnutrição em função do tempo ^1^.

Os efeitos fixos foram estimados para as variáveis IVS, ano, crise e a interação ano*crise. Utilizou-se o *ln* da população como variável *offset*. Quanto aos efeitos aleatórios do modelo, incluímos interceptos e coeficientes aleatórios, respectivamente, para as UF e o ano, especificando a matriz de covariância como não estruturada. Além disso, utilizamos métodos robustos para estimar as variâncias de modo a incorporar as dependências entre os erros das observações de uma mesma UF.

Considerando a complexidade da matriz de covariância e as grandezas do banco de dados, adotamos algumas estratégias para facilitar a convergência dos modelos. A primeira estratégia foi a recodificação da variável “ano” de 2008-2019 para 1-12. A segunda se deu por meio da construção do modelo final por etapas, com aumento da complexidade da parte fixa do modelo e da estrutura de covariância (partindo da permutável para a não estruturada). Assim, após a convergência de um modelo mais simples, seus parâmetros eram salvos e, então, utilizados como valores iniciais para o modelo subsequente.

As estimativas dos modelos foram apresentadas como risco relativo (RR) e seus respectivos intervalos de 95% de confiança (IC95%). As figuras com as tendências temporais de desnutrição foram plotadas utilizando as estimativas dos efeitos fixos (ajustadas para os efeitos aleatórios) preditas pelos modelos. Todas as análises foram realizadas no Stata/SE 13.0 (https://www.stata.com).

### Aspectos éticos

Por utilizar dados anonimizados e de domínio público, este estudo está dispensado da avaliação por um Comitê de Ética em Pesquisa com Seres Humanos.

## Resultados

**Figura 2:**
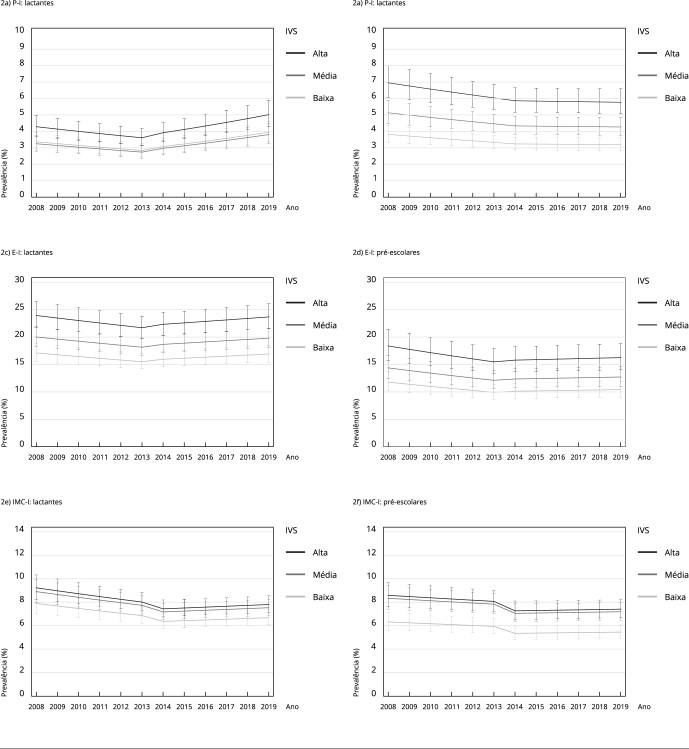
Tendência da prevalência de desnutrição (escore-Z de peso para idade, estatura para idade, e IMC para idade ≤ -2 DP) em crianças < 5 anos assistidas pelo Programa Bolsa Família categorizada pelo Índice de Vulnerabilidade Social das Unidades Federativas. Brasil, 2008-2019 *.

DP: desvio padrão; E-I: estatura para idade; IMC-I: índice de massa corporal para idade; IVS: Índice de Vulnerabilidade Social; P-I: peso para idade. * Excluídos os dados do Distrito Federal para lactentes entre 2008 e 2012, devido à ausência de dados no período.

A Tabela 1 apresenta os tamanhos de efeito para as variáveis independentes ano, crise, ano*crise e IVS em relação à tendência temporal de desnutrição no Brasil, segundo diferentes indicadores. Entre 2008 e 2019, estimou-se uma redução de 2 a 4% ao ano na prevalência de desnutrição nos lactentes e pré-escolares. No entanto, ao considerarmos o efeito da interação, foi observado que, de 2014 em diante, houve aumento no risco de desnutrição para ambos os grupos etários (lactentes: 3% a 8% ao ano; pré-escolares: 1% a 4% ao ano). De modo geral, o que se identificou foi que, após 2014, as tendências de desnutrição, que eram decrescentes, tornaram-se estacionárias, exceto para os indicadores de E-I e de P-I entre os lactentes, sobre os quais o efeito da crise foi intenso o suficiente para tornar as tendências ascendentes (Tabela 1; Figura 2).

**Tabela 1:** Risco relativo de desnutrição (escore-Z de peso para idade, estatura para idade e índice de massa corporal para idade ≤ -2 DP) em crianças < 5 anos assistidas pelo Programa Bolsa Família segundo ano (contínuo e categorizado), influência da redução do investimento em políticas de proteção social e Índice de Vulnerabilidade Social das Unidades Federativas. Brasil, 2008-2019.

Variáveis/Referência	Lactantes (0-23 meses)			Pré-escolares (24-59 meses)		
	P-I	E-I	IMC-I	P-I	E-I	IMC-I
	RR (IC95%)	RR (IC95%)	RR (IC95%)	RR (IC95%)	RR (IC95%)	RR (IC95%)
Ano						
2008-2019	0,96 (0,95; 0,98)	0,98 (0,97; 0,99)	0,97 (0,96; 0,98)	0,97 (0,95; 0,98)	0,96 (0,95; 0,97)	0,98 (0,98; 0,99)
Crise ^#^						
Pós-2014	0,62 (0,53; 0,72)	0,84 (0,78; 0,89)	0,73 (0,67; 0,79)	0,83 (0,73; 0,93)	0,79 (0,71; 0,89)	0,81 (0,75; 0,88)
Ano*crise ^##^						
2008-2019*pós-2014	1,08 (1,06; 1,10)	1,03 (1,02; 1,04)	1,03 (1,02; 1,05)	1,02 (1,00; 1,04)	1,04 (1,02; 1,05)	1,01 (1,00; 1,02)
IVS ^###^						
Média	0,96 (0,79; 1,17)	1,17 (1,05; 1,29)	1,12 (0,96; 1,32)	1,36 (1,17; 1,59)	1,22 (1,04; 1,42)	1,31 (1,13; 1,52)
Muito alta e alta	1,27 (1,02; 1,57)	1,39 (1,20; 1,62)	1,16 (1,02; 1,33)	1,88 (1,53; 2,11)	1,56 (1,25; 1,93)	1,35 (1,15; 1,59)

DP: desvio padrão; E-I: estatura para idade; IC95%: intervalo de 95% de confiança; IMC-I: índice de massa corporal para idade; IVS: Índice de Vulnerabilidade Social; P-I: peso para idade; RR: risco relativo de desnutrição (modelo linear generalizado multinível).

Nota: todos os modelos foram estatisticamente significativos ao nível p < 0,001 (teste de Wald).

^#^ Em relação ao período pré-2014;

^##^ Efeito da interação das variáveis ano e crise, visando estimar a influência da redução do investimento em políticas de proteção social nas tendências da prevalência de desnutrição nas crianças em situação de pobreza e extrema pobreza;

^###^ Em relação às Unidades Federativas com baixa vulnerabilidade social.

Quanto ao IVS, as análises indicaram consistentemente que crianças residentes em UF com média de vulnerabilidade social e muito alta e alta vulnerabilidade social apresentaram maior risco médio de desnutrição em relação aos seus pares provenientes de regiões com baixa vulnerabilidade social (Tabela 1). Considerando as diferenças mais marcantes entre os estados com baixa vulnerabilidade social e muito alta e alta vulnerabilidade social nas tendências de desnutrição (Figura 2), pode-se destacar o RR para a tendência de baixa estatura entre os lactentes (1,39; IC95%: 1,20; 1,62) e, entre os pré-escolares, as tendências de baixa estatura (1,56; IC95%: 1,25; 1,93) e magreza (1,35; IC95%: 1,15; 1,59) (Tabela 1).

As Tabelas 2 e 3 descrevem as variações das prevalências de desnutrição preditas pelos modelos, segundo o IVS, para lactentes e pré-escolares, respectivamente. Entre os lactentes, pode-se perceber mudanças nas tendências a partir da comparação dos períodos pré e pós-2014 com incrementos que vão de 3% para baixa E-I, no grupo de média vulnerabilidade social, à 36% para baixo P-I, no grupo de baixa vulnerabilidade social. Permaneceu com tendência de redução da prevalência apenas o indicador de baixa E-I no grupo de muito alta e alta vulnerabilidade social; no entanto, quando a variação do período pós-2014 (-2,5%) é comparada a do período pré-2014 (-8%), nota-se a tendência de manutenção dos valores. A mudança na tendência também pode ser percebida por meio dos incrementos, que chegam a 25,6% na comparação das prevalências de 2008 e 2019.

**Tabela 2:** Prevalência de desnutrição (escore-Z de peso para idade, estatura para idade e índice de massa corporal para idade ≤ -2 DP) em lactentes assistidas pelo Programa Bolsa Família agrupada pelo Índice de Vulnerabilidade Social das Unidades Federativas. Brasil, 2008, 2014 e 2019.

IVS/Ano	Lactantes (0-23 meses)					
	P-I		E-I		IMC-I	
	Prevalência (IC95%)	Variações (%)	Prevalência (IC95%)	Variações (%)	Prevalência (IC95%)	Variações (%)
Baixa						
2008	3,4 (2,6; 4,1)	-8,9 ^#^	16,3 (14,3; 18,4)	-3,3 ^#^	7,6 (6,4; 8,7)	-12,6 ^#^
2014	3,1 (2,7; 3,4)	36,0 ^##^	15,8 (14,4; 17,3)	16,2 ^##^	6,6 (5,6; 7,6)	25,3 ^##^
2019	4,1 (3,0; 5,3)	23,9 ^###^	18,4 (15,5; 21,2)	12,4 ^###^	8,3 (7,2; 9,4)	9,5 ^###^
Média						
2008	3,1 (2,8; 3,4)	0,4 ^#^	19,3 (17,1; 21,5)	-3,2 ^#^	9,6 (7,9; 11,2)	-15,3 ^#^
2014	3,1 (2,6; 3,7)	25,0 ^##^	18,7 (17,6; 19,8)	3,0 ^##^	8,1 (7,4; 8,8)	8,9 ^##^
2019	3,9 (3,1; 4,7)	25,6 ^###^	19,2 (18,1; 20,4)	-0,2 ^###^	8,8 (7,9; 9,7)	-7,8 ^###^
Muito alta e alta						
2008	4,5 (3,7; 5,3)	-11,7 ^#^	25,2 (22,3; 28,2)	-8,0 ^#^	9,4 (8,3; 10,6)	-16,0 ^#^
2014	4,0 (3,2; 4,7)	22,3 ^##^	23,2 (20,1; 26,4)	-2,5 ^##^	7,9 (7,4; 8,4)	15,7 ^##^
2019	4,8 (4,2: 5,4)	8,0 ^###^	22,7 (20,3; 25,0)	-10,2 ^###^	9,2 (8,5; 9,8)	-2,8 ^###^

DP: desvio padrão; E-I: estatura para idade; IC95%: intervalo de 95% de confiança; IMC-I: índice de massa corporal para idade; IVS: Índice de Vulnerabilidade Social; P-I: peso para idade.

^#^ Variação entre 2008 e 2014;

^##^ Variação entre 2014 e 2019;

^###^ Variação entre 2008 e 2019.

**Tabela 3:** Prevalência de desnutrição (escore-z de peso para idade, estatura para idade e índice de massa corporal para idade ≤ -2 DP) em pré-escolares assistidos pelo Programa Bolsa Família agrupada pelo Índice de Vulnerabilidade Social das Unidades Federativas. Brasil, 2008, 2014 e 2019.

IVS/Ano	Pré-escolares (24-59 meses)					
	P-I		E-I		IMC-I	
	Prevalência (IC95%)	Variações (%)	Prevalência (IC95%)	Variações (%)	Prevalência (IC95%)	Variações (%)
Baixo						
2008	3,7 (3,0; 4,5)	-18,7 ^#^	11,8 (9,9; 13,6)	-14,4 ^#^	6,3 (5,2; 7,4)	-8,9 ^#^
2014	3,0 (2,7; 3,4)	5,1 ^##^	10,1 (9,2; 11,0)	8,5 ^##^	5,7 (5,0; 6,5)	12,5 ^##^
2019	3,2 (2,6; 3,7)	-14,5 ^###^	11,0 (9,6; 12,3)	-7,0 ^###^	6,5 (5,4; 7,5)	2,5 ^###^
Média						
2008	4,8 (4,1; 5,5)	-9,4 ^#^	13,9 (11,5; 16,3)	-1,6^#^	8,5 (7,4; 9,7)	-10,3 ^#^
2014	4,3 (3,8; 4,9)	-4,4 ^#^	13,6 (9,8; 17,5)	-3,5 ^##^	7,6 (7,0; 8,3)	3,9 ^##^
2019	4,1 (3,6; 4,7)	-13,4 ^###^	13,2 (11,8; 14,5)	-5,1 ^###^	7,9 (7,3; 8,6)	-6,8 ^###^
Muito alta e alta						
2008	7,2 (5,9; 8,5)	-18,3 ^#^	20,3 (16,9; 23,8)	-16,0 ^#^	9,0 (7,9; 10,1)	-16,6 ^#^
2014	5,9 (4,8; 7,0)	7,1 ^##^	17,1 (13,6; 20,5)	-5,0 ^##^	7,5 (6,6; 8,4)	10,7 ^##^
2019	5,5 (4,6; 6,4)	-24,1 ^###^	16,2 (13,4; 19,0)	-20,2 ^###^	8,3 (7,7; 9,0)	-7,6 ^###^

DP: desvio padrão; E-I: estatura para idade; IC95%: intervalo de 95% de confiança; IMC-I: índice de massa corporal para idade; IVS: Índice de Vulnerabilidade Social; P-I: peso para idade.

^#^ Variação entre 2008 e 2014;

^##^ Variação entre 2014 e 2019;

^###^ Variação entre 2008 e 2019.

Entre os pré-escolares (Tabela 3), apesar da tendência de redução da prevalência de desnutrição ao se considerar todo o período, nota-se comportamento semelhante ao observado no grupo de lactentes, com variações positivas ou tendência de estabilização após 2014.

## Discussão

O objetivo deste estudo foi analisar a tendência temporal entre 2008 e 2019 da prevalência de desnutrição em lactentes e pré-escolares assistidos por aquele que foi, até sua extinção em 2021, o maior programa de transferência condicional de renda do mundo em termos de financiamento e cobertura ^26^^,^^27^. Nossas análises identificaram que o ano de 2014 foi um ponto de inflexão nas tendências da desnutrição infantil, momento em que se tornaram, de modo geral, estacionárias para pré-escolares e ascendentes para lactentes.

Considerando que a redução da prevalência de desnutrição infantil até o ano de 2014 é produto do sucesso das políticas compensatórias ^9^^,^^10^^,^^11^, esperava-se que os indicadores continuassem regredindo nos anos seguintes até que a desnutrição infantil em crianças de baixa renda deixasse de ser um problema de saúde pública. No entanto, o comportamento de estabilização dos indicadores em patamares acima do esperado ou de mudança de sentido para os indicadores que se apresentavam próximos dos valores considerados ideais, não são produtos do acaso. De acordo com a hipótese especificada no modelo, foi uma mudança de conjuntura política, econômica e social que repercutiu na prevalência de desnutrição infantil a partir de 2014.

Tais achados corroboram a hipótese levantada por Vasconcelos et al. ^22^. Nesse trabalho, os autores especularam que a redução da prevalência de excesso de peso entre lactentes e pré-escolares assistidos pelo Programa Bolsa Família não foi resultado de políticas públicas de enfrentamento da obesidade infantil, aumentando o contingente populacional de crianças com peso saudável, mas sim produto das políticas de austeridade fiscal, resultando no aumento da desnutrição infantil.

Da transformação do superávit primário em resultado primário negativo e sua repercussão sobre a elevação da dívida pública. Da inflação e dos eventos de corrupção revelados pela Operação Lava-Jato, emergiu uma nova conjuntura favorável para o direcionamento das políticas públicas para o sentido das privatizações, do ajuste fiscal, da flexibilização da legislação trabalhista e da revisão das políticas sociais ^28^^,^^29^. Como resultado, houve redução de 42% nos investimentos públicos em despesas primárias e em áreas estratégicas, como saúde, educação e assistência social ^30^, impactando negativamente, ainda mais nas regiões mais pobres ^29^.

Tal redução dos investimentos públicos associada à *Emenda Constitucional nº 95* (EC 95), que limita o crescimento das despesas primárias ao índice de inflação por 20 anos, tem promovido um significativo retrocesso das políticas públicas voltadas para a garantia da condição de bem-estar social, pois os valores investidos no final desse período representarão um percentual do Produto Interno Bruto (PIB) inferior ao investido em 2006 ^30^^,^^31^.

Os resultados deste estudo demonstram que, como efeito de curto prazo, tais políticas já promovem a diminuição no potencial de crescimento linear de crianças em situação de vulnerabilidade, representando, assim, não mais uma ameaça, mas a ruptura com os pactos internacionais e com o sucesso das políticas de suporte social no combate à fome e à desnutrição ^23^^,^^32^. Além disso, a redução de investimentos sociais, especialmente em programas voltados para a proteção e promoção da infância, poderá causar, como efeitos de médio e longo prazos, o comprometimento da produtividade pela redução do capital humano e, consequentemente, o aumento do desemprego e subemprego para níveis ainda mais alarmantes ^1^^,^^2^^,^^4^^,^^32^.

No entanto, com o início da pandemia de COVID-19 e as mudanças estruturais decorrentes da ação, inação ou de sua negação pelo Poder Público, o cenário da fome e da desnutrição infantil deverá ser agravado, uma vez que suas repercussões socioeconômicas impactam e comprometem particularmente as pessoas em situação de pobreza ^33^.

Dados do banco mundial sugerem que a pandemia da COVID-19 produziu um impacto sem precedentes no aumento da pobreza mundial ^34^. Além disso, os sistemas alimentares já estão sendo altamente impactados, ocasionando o aumento da insegurança alimentar em nível global ^17^.

No Brasil, após 2013, a prevalência de insegurança alimentar grave passou a apresentar uma tendência ascendente (2013-2018: +1,6p.p. [pontos percentuais]; 2018-2020: +3,2p.p.; 2020-2021/22: +6,5p.p.), chegando, em 2020 e 2021, ao patamar de 15,5% (33 milhões de pessoas). No entanto, essa situação se torna mais alarmante ao observarmos os dados da insegurança alimentar grave em domicílios com menores de 10 anos; nessas residências, as prevalências de insegurança alimentar moderada e grave foram, respectivamente, 18,9% e 18,1% ^17^.

O feito de exponenciar a disseminação da fome, especialmente no último período (1 ano e 4 meses) é produto da fragilização das estruturas de suporte do estado de bem-estar social e do desequilíbrio econômico que resultou no aumento do preço dos alimentos. Segundo o Índice Nacional de Preços ao Consumidor Amplo (IPCA) ^35^, que mede a inflação de produtos e serviços comercializados no varejo, entre julho de 2021 e junho de 2022, houve um aumento de 11,89% nos gastos gerais das famílias brasileiras, entre os quais o maior ocorreu no componente alimentos e bebidas (13,93%), impactando principalmente o acesso à alimentação das famílias empobrecidas.

Nesse cenário de ameaças gravíssimas à infância, tem-se, ainda, o fechamento das escolas durante boa parte da pandemia, gerando um duplo impacto para o crescimento e desenvolvimento infantil. Se por um lado a participação no Programa Nacional de Alimentação Escolar (PNAE) foi limitado, comprometendo o acesso a uma alimentação adequada e saudável, por outro, o direito à educação (pré-escolar e escolar) de crianças em situação de vulnerabilidade social e econômica foi duramente restringido, especialmente pela impossibilidade de acesso aos conteúdos remotos ^36^.

Outro componente importante identificado foi o efeito estatisticamente significativo do IVS das UF na tendência de desnutrição infantil, indicando desigualdades na distribuição da desnutrição no Brasil. Diante da magnitude e da complexidade dessas desigualdades ^37^^,^^38^^,^^39^^,^^40^^,^^41^, torna-se evidente a necessidade de suspender a EC 95 e reorganizar as políticas públicas voltadas para a efetivação da Lei Orgânica de Segurança Alimentar e Nutricional, a fim de reduzir os efeitos das crises política, econômica e sanitária na desnutrição infantil e proteger as futuras gerações.

Assim, considerando o papel do Estado como promotor de bem-estar, há a necessidade de avançar em agendas intersetoriais que articulem não apenas a retomada dos investimentos em serviços de saneamento básico, APS e de educação, mas também políticas macroeconômicas de geração de empregos e que promovam sistemas alimentares saudáveis e sustentáveis, para permitir o retorno das tendências de erradicação da desnutrição e de vulnerabilidades que impedem os mais pobres de vislumbrar a superação de suas dificuldades ^2^.

Este estudo apresenta algumas limitações. Existe um grau de incerteza, ainda indeterminado, quanto à precisão dos dados antropométricos disponíveis no SISVAN, uma vez que essas informações são geradas a partir de coletas realizadas como parte das rotinas dos serviços de saúde por um amplo número de profissionais e com diferentes níveis de treinamento em antropometria; além disso, outras potenciais fontes de erro podem estar na manutenção e calibração dos equipamentos antropométricos e na digitação dos dados (prontuário eletrônico ou inserção manual pelas secretarias municipais de saúde). Por outro lado, é importante considerar que essas informações representam estatísticas oficiais do Estado Brasileiro. Além disso, a elevada cobertura do SISVAN para os dados antropométricos, o número de observações por UF por ano, os limites dos intervalos de confiança e a consistência das informações sugere um baixo nível de incerteza quanto à exatidão de nossas estimativas, especialmente em relação à dinâmica da tendência temporal da prevalência de desnutrição em crianças assistidas pelo Programa Bolsa Família.

## Conclusões

No período analisado (2008-2019), houve redução na prevalência de desnutrição infantil até meados de 2013, quando se estabeleceu um ponto de inflexão, tornando as tendências estacionárias ou ascendentes. Tais trajetórias corroboram a hipótese deste trabalho, a qual afirma que as crises política e econômica, e as respostas governamentais a essas crises, provocaram impacto negativo sobre o estado nutricional de crianças em situação de pobreza e extrema pobreza.

Dessa conclusão, contextualizada no cenário da pandemia de COVID-19, é possível extrair duas implicações para gestores públicos. A primeira diz respeito à necessidade de retomar o monitoramento ativo da desnutrição infantil, especialmente em regiões e territórios com alta prevalência de famílias em situação de pobreza e pobreza extrema. A segunda, e mais importante, refere-se à urgência na reversão das políticas de austeridade fiscal adotadas pelo Governo Federal, no fortalecimento do SISAN e na implementação de estratégias que possibilitem a superação da pobreza em curto prazo, como a efetivação da renda básica da cidadania, prevista na *Lei nº 10.835/2004*^42^.
